# Crash Frequency Analysis Using Hurdle Models with Random Effects Considering Short-Term Panel Data

**DOI:** 10.3390/ijerph13111043

**Published:** 2016-10-26

**Authors:** Feng Chen, Xiaoxiang Ma, Suren Chen, Lin Yang

**Affiliations:** 1 Department of Traffic Engineering and Key Laboratory of Road & Traffic Engineering of the Ministry of Education, Tongji University, 4800 Cao’an Road, Shanghai 201804, China; fengchen@tongji.edu.cn; 2 Department of Civil & Environmental Engineering, Colorado State University, Fort Collins, CO 80523, USA; xiaoxiang.ma@colostate.edu; 3 College of Transportation Engineering, Tongji University, 4800 Cao’an Road, Shanghai 201804, China; yanglin0827@126.com

**Keywords:** daily crash frequency, short-term driving environment, panel data, hurdle negative binomial, random effect

## Abstract

Random effect panel data hurdle models are established to research the daily crash frequency on a mountainous section of highway I-70 in Colorado. Road Weather Information System (RWIS) real-time traffic and weather and road surface conditions are merged into the models incorporating road characteristics. The random effect hurdle negative binomial (REHNB) model is developed to study the daily crash frequency along with three other competing models. The proposed model considers the serial correlation of observations, the unbalanced panel-data structure, and dominating zeroes. Based on several statistical tests, the REHNB model is identified as the most appropriate one among four candidate models for a typical mountainous highway. The results show that: (1) the presence of over-dispersion in the short-term crash frequency data is due to both excess zeros and unobserved heterogeneity in the crash data; and (2) the REHNB model is suitable for this type of data. Moreover, time-varying variables including weather conditions, road surface conditions and traffic conditions are found to play importation roles in crash frequency. Besides the methodological advancements, the proposed technology bears great potential for engineering applications to develop short-term crash frequency models by utilizing detailed data from field monitoring data such as RWIS, which is becoming more accessible around the world.

## 1. Introduction

Traffic crashes cause a lot of occupancy injury and serious congestion on the road systems around the world. Knowledge about critical contributing factors and the prediction of crash risk are critical to developing next-generation crash prevention technologies. In the recent review studies conducted by Lord and Mannering [1] as well as Mannering and Bhat [2], some methodology challenges encountered in current crash frequency studies were summarized, including temporal and spatial correlation, time-varying explanatory variables, and omitted-variables bias. Most of the existing crash frequency modeling methods use aggregated data in extended time scales (e.g., yearly or monthly), instead of fine time scales (e.g., hourly, daily) containing detailed time-varying information. The extended scales and aggregated variables lead to some limitations as summarized in references [1,2]. For example, some important explanatory variables in crash frequency models sometimes change quickly over time, such as weather, road surface conditions and traffic flow. By adopting extended time scales, some critical information over time of those important influencing variables (e.g., weather condition, traffic speed) is inevitably missing [1]. The crash frequency prediction models derived from the aggregated data may fail to capture important time-varying and/or space-varying information and introduce errors into model results due to unobserved heterogeneity [3,4].

However, when data on smaller time scales (e.g., hours, days, rather than years) is adopted in crash frequency models, two major associated methodological challenges arise: (1) time-specific heterogeneity (e.g., micro-climates such as a snow storm, or temporal effects such as the weekend, which may influence the crash risk of the road segments in the same area at the same time); and (2) the preponderant portion of non-crash observations (more zero observations under fine time scales). Specifically, when the scales get smaller, the same road segment and/or the same time period may have multiple observations, which may be sharing unobserved effects because of the correlation over time and/or space [1]. The second challenge associated with short-term data is excess zeros. Because of the fine time scales, there may be tremendous observations with no crash which indicate a big portion of zero counts. Consequently, excessive zeros need to be appropriately taken care of if a short-term crash frequency model is to be developed.

With the wide applications of ITS (Intelligent Transport System) in recent years, real-time traffic and weather monitoring data becomes more conveniently obtainable on many road systems unprecedentedly. By taking advantage of these real-time data, many researchers have attempted to develop short-term crash prediction models, which primarily studied crash risk or likelihood [5,6,7,8,9,10,11,12,13,14,15,16,17,18]. There is, so far, rarely reported studies on the direct prediction of short-term crash frequency and, therefore, the associated technical challenges remain unsolved. In modern social science, panel data models have been widely used when considering data with both spatial- and time-varying characteristics, partly because they are capable of addressing the heterogeneity of the individuals. Being applied in the traffic engineering field, fixed effect or random effect Poisson and negative binomial models have been adopted for crash prediction studies recently [19,20,21]. Although they shed light on dealing with the first challenge (i.e., time-specific heterogeneity) as summarized above, these studies were mostly about extended time period modeling and only dealt with heterogeneity in monthly or yearly repeated observations. 

As extensions of the Poisson and negative binomial models, Zero-inflated Poisson and Zero-inflated negative binomial (ZINB) models have sometimes been used to handle mass zero counts in crash data [22,23,24,25]. A previous study by the authors examined hourly crash frequency using the ZINB model and showed that ZINB is capable of addressing preponderance zeroes in crash data [26]. However, zero-inflated models used for crash prediction also received some criticisms because of the structural-zero assumption [27,28]. As an alternative to zero-inflated models, hurdle models have some inherent advantages on model assumptions. By relaxing the structural zero assumption, hurdle models assume that all zeroes in the crash data are sampling zeroes [29,30,31,32]. In contrast to the structural zero assumption presuming an inherently safe condition with no crashes, sampling zero assumption implies that all segments have crash potential and the zero state does not remain permanently on any road segment [29,30,31,32]. Existing crash studies using hurdle models [29,30,31,32,33] were primarily developed based on cross-sectional data (as opposed to panel data) and did not consider random effects. These studies ignore temporal and spatial correlations, which, however, are critical to short-term panel data. In light of the above discussion, this study explores the development of the random effect hurdle models for crash frequency prediction on fine time scales for the first time. The proposed model considers the correlation of observations, the unbalanced panel-data structure, and dominating zero observations. 

## 2. Materials and Methods

In the following study, we select a mountainous portion of the interstate highway I-70 corridor in Colorado for a demonstration. The I-70 mountain corridor is a typical mountainous highway that features steep slopes and sharp curves. Moreover, due to high elevation, I-70 often experiences inclement and fast changing weather conditions, such as snow, rain, and wind. In preparation for the following data analysis, an integrated crash database is first established, which contains information on crash data, road geometry, real-time weather, road surface conditions and traffic flow information. A portion of I-70 with a length of 56.06 miles (mile marker 195.26 to 251.32) is chosen for the study. The portion of I-70 is first split into homogeneous roadway segments, which are based on splitting criteria concerning the change of lane width, shoulder type, median type, speed limit and pavement conditions. One hundred homogeneous roadway segments are generated with an average length around one mile, including 52 eastbound and another 48 westbound segments. Because of the typical mountainous terrain along with the fast-changing nature of adverse weather on I-70, detailed weather factors in a short time period become very critical in terms of influencing road safety as compared to many other time-constant ones. Four types of crash-related data from this 56.06 mile section are included in this study: (1) traffic crash data; (2) highway geometry data (including pavement characteristics); (3) real-time road surface and weather condition data; (4) real-time traffic data. The final combined database contains daily distributions of crashes, traffic, road surface and weather condition data for roadway segments on both driving directions. By combining short-term data from different sources, we can perform a more insightful and comprehensive study on critical influencing factors. 

The traffic crash data, provided by the Colorado State Patrol (CSP, Department of Public Safety, Lakewood, CO, USA), spans from January 2010 to December 2010. Daily records of crash data are generated for each segment according to the occurrence time of each crash and then the panel data structure is established accordingly. The roadway geometry data is from the Roadway Characteristics Inventory (RCI) operated by the Colorado Department of Transportation (CDOT, Denver, CO, USA). The dataset from RCI provides detailed roadway design features and pavement characteristics, including speed limit, segment length, number of lanes, lane width, horizontal curvature, grade, shoulder width, median types, median width, international roughness index, rut depth, etc. Temporal dummy variables for each day, such as month, day of the week, etc. represent the temporal distribution of crash frequency. However, the temporal dummy variables were found to be not significant in the models.

In addition to these roadway characteristics commonly used in accident modeling, this study also incorporates short-term traffic and weather/surface data provided by the road weather information system (RWIS). There are 24 traffic stations along the study area on the I-70 corridor providing real-time monitoring data of traffic speed and volume. The real-time traffic data, which were originally recorded in 2 min intervals, are processed into daily records to facilitate the following study. For example, the daily average, standard deviation and minimum of traffic speed and the daily average, standard deviation and maximum of traffic volume have been considered in the statistical models as possible influence factors. 

Seven weather stations are installed along the study area, providing motorists with real-time weather conditions. Moreover, the detailed weather and road surface condition data were provided by these weather stations in this study. In the CDOT database, precipitation status types include None, Rain, Snow, Hail and Unknown type precipitation. The real-time weather and surface condition data was collected every 20 min. In addition, the daily average and minimum value of the visibility have also been considered as the candidates of the influence factors. In order to generate the daily records for precipitation status or road surface condition, the ratio of precipitation status or road surface condition in the day road surface condition is adopted. For example, the ratio of the wet road surface in the day means the number of the road surface condition records as wet road divided by the number of all the road surface condition records within that day. Other weather variables, such as temperature, precipitation amount and humidity the average, minimum and maximum values of these variables have been chosen as the candidates of influence factors. Time-varying data from the closest weather and traffic stations are assigned to each roadway segment.

Due to sensor malfunctions, sometimes real-time data records leave “empty” windows (i.e., no data for one or several sensors at some time). After discarding those data records with missing values, a total of 29,462 records were generated. A total of 783 crashes occurred in these records. As a result, the panel data structure becomes unbalanced, posing additional challenges on following model development. A summary statistics of the explanation variables are shown in Table 1.

In typical crash frequency models for a yearly or monthly data structure, observations are typically assumed to be independent. This assumption is likely violated with repeated measures in short-term time periods, such as crash counts during different time periods at the same site or those between different road segments at the same time instant. Certain correlations among those repeated observations may occur and random effect models are usually adopted to deal with this issue. In order to identify the most appropriate model for the study, we compare some possible candidate models including random effect Poisson (REP), random effect negative binomial (RENB), random effect hurdle Poisson (REHP) and random effect hurdle negative binomial (REHNB).

The total number of observations is denoted as *N*,

(1) $$N=\sum_{i=1}^{I}t_{i}=\sum_{t=1}^{T}i_{t}$$

where *t* = *1*, . . . , *T*, and *i_t_* is the number of different site observations in time period *t*; *i* = *1*, . . . , *I*, and *t_i_* is the number of repeated observations in site *i*. Under balanced panel data structure, *t_i_* is the same for all sites or *i_t_* is the same for all time periods. Since the short-term weather, road surface, and traffic data was not all continuous, *t_i_* and *i_t_* are different, and thus for this data the data structure is actually unbalanced panel data.

We start with the random effect Poisson model as an example of random effect models, which are applied to panel data. Letting $n_{it}$ be the crash frequency on roadway segment (*i*) during period (*t*), the random effect Poisson model is shown as:

(2) $$P(n_{it})=\frac{\exp(-\lambda_{it})\lambda_{it}^{n_{it}}}{\Gamma (n_{it}+1)}$$

where $P\left(n_{it}\right)$ is the probability of *n* crashes happening on the highway segment (*i*) in time period (*t*) and $\lambda_{it}$ is the Poisson parameter which equals to the expected mean value of $n_{it}$ ($E\left(n_{it}\right)$):

(3) $$\lambda_{it}=\exp(\beta x_{it}+\sigma_{i})$$

where $\lambda_{it}$ is the Poisson parameter, β is the vector of regression coefficients, and $x_{it}$ is the vector of covariates defining crash frequency on roadway segment (*i*) during predetermined time period (*t*); $\sigma_{i}$ is the random effect parameter with independent normal distributions, i.e., $\sigma_{i}\sim N(0,\varphi_{\sigma_{i}}^{2})$ ($\varphi_{\sigma_{i}}$ is the standard deviation of $\sigma_{i}$).

The main limitation of the Poisson model is that it restricts the mean and variance of the distribution being equal [24]. The possibility of over-dispersion (having variance exceeding the mean) will result in biased coefficient estimates. To relax this constraint facing by the Poisson model, a negative binomial distribution is usually adopted [22,23,34,35]. The random effect negative binomial model is derived from Equation (2) by adding a Gamma-distributed error term such that,

(4) $$\log(\lambda_{it})=\beta x_{it}+\varepsilon_{it}+\sigma_{i}$$

where $\sigma_{i}$ is the random effect parameter with independent normal distributions and $\varepsilon_{it}$ is the Gamma-distributed error term, and this addition allows the variance to differ from the mean [24]:

(5) $$\mathrm{Var}[n_{it}]=E[n_{it}][1+\alpha E[n_{it}]]=E[n_{it}]+\alpha E{\left[n_{it}\right]}^{2}$$

where *α* is an additional estimable coefficient.

The negative binomial distribution is shown as:

(6) $$P(n_{it})=\frac{\Gamma ((1/\alpha )+n_{it})}{\Gamma (1/\alpha )\Gamma (n_{it}+1)}{\left(\frac{1/\alpha }{(1/\alpha )+\lambda_{it}}\right)}^{1/\alpha }{\left(\frac{\lambda_{it}}{(1/\alpha )+\lambda_{it}}\right)}^{n_{it}}$$

Considering the excess zeros issue, the hurdle Poisson model with random effects has two different states (zero state and Poisson state), which are shown as:

(7a) $$n_{it}=0$$

with probability

(7b) $$q_{it}$$

(8a) $$n_{it}=y\;(y=1,2,\ldots )$$

with probability

(8b) $$(1-q_{it})\frac{e^{-\lambda_{it}}{\lambda_{it}}^{n_{it}}}{(1-e^{-\lambda_{it}})n_{it}!}$$

where

(8c) $$\lambda_{it}=exp(\beta x_{it}+\sigma_{i})$$

(8d) $$\mathrm{logit}(q_{it})=\ln(\frac{q_{it}}{1-q_{it}})=\theta A_{it}+\psi_{i}$$

$A_{it}$ and $x_{it}$ are the covariates with θ and β as their coefficient vectors; σ and ψ are the random effect parameters for zero state and Poisson state with independent normal distributions, i.e., $\sigma \sim N\left(0,\varphi_{\sigma }^{2}\right)$ and $\psi \sim N\left(0,\varphi_{\psi }^{2}\right)$ ($\varphi_{\sigma }$ and $\varphi_{\psi }$ are the standard deviations of σ and ψ).

Moreover, the hurdle negative binomial model with random effects is shown as

(9a) $$n_{it}=0$$

with probability

(9b) $$q_{it}$$

(10a) $$n_{it}=y\;(y=1,2,\ldots )$$

with probability

(10b) $$(1-q_{it})\left(1-\frac{1}{{\left(1+\alpha \mu_{it}\right)}^{\left(1/\alpha \right)}}\right)\left(\frac{\Gamma ((1/\alpha )+n_{it})}{\Gamma (1/\alpha )n_{it}!}\right)\left(\frac{{\left(\alpha \mu_{it}\right)}^{n_{it}}}{{\left(1+\alpha \mu_{it}\right)}^{n_{it}+(1/\alpha )}}\right)$$

where

(10c) $$u_{it}=(1/\alpha )[(1/\alpha )+\lambda_{it}]$$

All the parameters in Equations (7)–(10) share the same definitions as those defined above for RENB and REHP models.

Several tests can be used to verify the presence of over-dispersion and the causation of zero counts on over-dispersion, such as the dispersion parameter, *Z*-score and Lagrange multiplier (LM) tests, Vuong’s test [30], among others. The over-dispersion parameter α of RENB is significant with a *t*-statistic of 6.72, which indicates the appropriateness of the RENB model over the REP model and the presence of over-dispersion. The corresponding *p* values for the *Z*-score and LM tests (for test calculation details, please refer to [30]) are all less than 0.0001, which also imply the existence of over-dispersion in the data. 

With the short-term panel data structure, it is apparent that there are a high number of zero crash observations; however, it is still not clear whether the random effect hurdle model is truly more statistically appropriate than its counterparts (REP and RENB models). Vuong’s test has often been used for comparing non-nested models and different count data models. Here it is used to compare REHNB vs. REP models, and REHNB vs. RENB models. Vuong [36] proposed a *t*-statistic–based test which first computes $m_{it}$: as a transition parameter:

(11) $$m_{it}=\ln\left(\frac{f_{1}(y_{it}|X_{it})}{f_{2}(y_{it}|X_{it})}\right)$$

where $f_{1}(y_{it}|x_{it})$ could be the probability density function of the REHNB or REHP model and $f_{2}(y_{it}|x_{it})$ could be the probability density function of the RENB or REP model.

Then Vuong’s statistic is tested as [23,36],

(12) $$V=\frac{\bar{m}\sqrt{N}}{S_{m}}$$

where $\bar{m}$ and $S_{m}$ are the mean and the standard deviation of $m_{it}$; N is the sample size. The REHNB model is statistically preferred because the absolute value of *V* is bigger than 1.96 (−26.4 vs. RENB and −25.9 vs. REP). 

In addition, the Akaike information criterion (AIC) has frequently been used for model selection which is shown in Equation (13).

(13) AIC=-2(lnL-p)

where L is the likelihood value of the model; p is the number of parameters of the model. 

Among different models developed with the same dataset, the one with the lower AIC value is usually preferred. The AIC value of REHNB is smaller than those of the REHP, RENB and REP models (6320.9 vs. 6342.7, 6320.9 vs. 6357.4 and 6320.9 vs. 6497.9), which means that the REHNB model is favored over the RENB and REP models according to Hilbe’s AIC rule of thumb [30,37,38]. These results indicate that the presence of over-dispersion in the short-term crash data is due to both excess zeroes and unobserved heterogeneity. Without considering the random effect, most of the research [29,30,31,32] about crash frequency modeling using hurdle models found that hurdle negative binomial models are not convergent or not preferred. For example, Hosseinpour et al. [30] have found that the hurdle Poisson model instead of the hurdle negative binomial model was the best one among different models being considered in terms of comparative measures.

## 3. Results and Discussion

The REHNB model results are presented in Table 2. The variables in Table 2 for both the negative binomial state and the zero state are statistically significant at a 90% confidence level. The results show that many factors significantly influence the crash frequency on I-70, including time-varying variables (e.g., daily minimum visibility and daily traffic volume) and site-varying variables (e.g., inside shoulder width indicator and the indexed value of the international roughness index). These factors include those of road, environmental, and traffic characteristics. Temporal characteristics, such as the weekend indicator, holiday indicator and month indicators are found to be not significant. The reason why month indicators are not significant is perhaps that we have already accounted for the road surface and weather conditions in the model.

The random effect parameter $\psi_{i}$ is significant at the 99.9% level and $\sigma_{i}$ is significant at the 97.2% level, which indicates that it is reasonable to adopt random effect specification (the *t*-statistic for $\psi_{i}$ is 8.89 and for $\sigma_{i}$ is 2.23). The likelihood test between the REHNB and hurdle negative binomial also shows the significance of the random effect with the *p* value of the likelihood test less than 0.001 (the detailed calculation and results are not included here to save space). The over-dispersion parameter α is 662.87 for the REHNB model, which indicates that the REHNB model is preferred. Therefore, for the present study, the REHNB model is found to be the most preferred one. To save space, only the REHNB model estimation results are demonstrated in the following. If the parameter coefficient is positive in the zero state, the probability in the zero state will increase while the mean value of the predicted crash count will decrease when the parameter gets larger. Moreover, the predicted mean of the crash count will increase if the parameter coefficient is positive in the negative binomial state. 

### 3.1. Traffic Characteristics

With regard to traffic speed, the daily average speed gap (difference between the speed limit and daily average traffic speed) is used to present the gap between the speed limit and the daily average traffic speed. We find that crash frequency will increase with a bigger daily average speed gap (a negative coefficient in the zero state). Because the raw speed data was truncated for any traffic speed higher than the speed limits, the recorded traffic speed will not be higher than the speed limit. Therefore, a larger daily average speed gap essentially presents that congestion perhaps occurs during this day. These results show that the occurrence of congestion is positively related to the crash frequency in the study area. Yu and Abdel-Aty [17] also made similar observations that an increase in the multi-vehicle crashes likelihood is associated with congested conditions at the downstream.

As for daily traffic volume, higher ones decrease the possibility of road segment being in the zero state (a negative coefficient), which means that the model may be pushed to the negative binomial state with a higher daily traffic volume and the crash frequency will increase accordingly. Some other studies have made similar conclusions (e.g., [39]).

### 3.2. Weather/Surface Characteristics

It is found that the ratio of wet road surface in the day brings a higher crash frequency (negative coefficient in the zero state). In addition, some other inclement weather and road surface conditions are also found to contribute to the increase of the crash frequency, including the ratio of the chemical wet road surface in the day, the ratio of icy warning road surface in the day, and the ratio of snowing status in the day. The above results on the typical mountainous highway I-70 highlight the significant safety threats under adverse weather and road surface conditions on mountainous highways as well as the need for further studies. Given the high crash risks of mountainous highways, this finding may also shed some light on some potential mitigation efforts to reduce crash counts. In addition, daily minimum visibility is chosen as a variable in the final model based on the best model fit (compared with daily average visibility), and a higher daily minimum visibility is found to push road segments to the zero state, which indicates that better visibility conditions lower the probability of a higher daily crash frequency. This finding has been confirmed by some short-term crash probability modeling work (e.g., [14,18,39]). 

### 3.3. Road Characteristics

Turning to parameters of roadway geometric characteristics, the larger length of the highway segment leads to a higher crash frequency in both the negative binomial state and the zero state along I-70, which implies that road segments with a longer segment length will perhaps have more crashes. 

The RUTI (rut index) variable represents the remaining service life for the rut in the CDOT database. For example, the value of 100 indicates a 0.15 inch rut or less. By using different thresholds of RUTI, and based on the best model fit, a dummy variable is adopted which is named as the long remaining service life of the rut indicator (one if the value of RUTI is higher than 99, zero otherwise). A longer remaining service life of the rut contributes to a higher crash frequency (with a positive sign in the negative binomial state) according to the modeling results. This is possibly because the driver inclines to be more careful on deep, rutted road segments. Anastasopoulos and Mannering [40] found that under excellent rut conditions, the majority of the road segments cause a decrease in the crash occurrences condition while other road segments still show the opposite. In addition, it is interesting to find that a higher international roughness index (lower values equal rougher roads) results in a lower crash frequency. It is known that road surface roughness can considerably influence vehicle performance and maneuverability. The results suggest that although rut may make drivers more alert, road surface conditions still need to be maintained in order to promote traffic safety. The inside shoulder width indicator is positive in the negative binomial state which indicates that roadway segments with wider inside shoulders result in a higher crash frequency. Anastasopoulos and Mannering [40] found that the same inside shoulder width indicator has a random distribution and it has a mostly negative effect on crash frequency. The different phenomena observed in the two studies may be attributable to different driving behaviors on mountainous and non-mountainous highways and more insightful investigations are still needed in this regard.

## 4. Conclusions

Given the criticism against ZINB in safety studies, the present study develops random effect hurdle negative binomial and Poisson models to study crash frequency on fine time scales as a complementary study to our prior research [26]. Firstly, random effect models including REHNB, REHP, RENB and REP are established to study the daily crash frequency with the panel data. Four candidate models are developed and compared in order to identify the most appropriate model for the I-70 mountainous highway. According to the author's knowledge, random effect hurdle models are used for short-term crash frequency modeling for the first time. Secondly, in addition to the methodological contribution, this study also demonstrates a promising engineering technique of developing short-term crash frequency models based on field monitoring data. Detailed data with refined temporal and spatial distributions, including crash record, road design, time-varying weather conditions, road surface conditions and traffic conditions from I-70 in Colorado, are adopted in the study. Without significant additional investments in data collection equipment, this method can potentially be applied to other highways in the country or around the world. Last but not least, because of the rich information of the datasets, this study can adopt short-term data with comprehensive coverage of various variables, and therefore provides insights for crash frequency prediction in terms of traffic characteristics, environmental characteristics, and road characteristics. Some key findings are summarized as follow.

1. In addition to site-varying factors (e.g., inside shoulder width indicator and the indexed value of the international roughness index), time-varying factors (e.g., daily traffic volume and road surface conditions) also have a significant influence on the crash frequency on interstate highway I-70. It is worth noting that many different types of road surface conditions can considerably influence the crash frequency.
2. The results of several different statistical tests show that over-dispersion exists in the short-term data. In addition, the preference of the REHNB models indicated that the over-dispersion arises because of both unobserved heterogeneity and excess zeroes. Vuong’s test is conducted for two pairs of candidate models: REHNB versus REP and REHNB versus RENB. The test results also confirm the above finding and the REHNB model is found to be the most suitable model for I-70 according to Vuong’s test and AIC. These findings highlight the importance of handling both unobserved heterogeneity and excess zero issues in short-term data, as well as the appropriateness of the random effect hurdle negative binomial model for this type of data.
3. This paper explores developing new short-term crash frequency models (e.g., daily) using real-time traffic flow, weather and road surface condition data. Such a study has some potential for further traffic safety improvements. The models and the findings in this paper may open a door toward consequence-based highway design, active traffic management strategies, and intelligence-based law enforcement interventions in the future.

## Figures and Tables

**Table 1:** Summary statistics of the data for observations.

Variable	Mean	Standard Deviation	Minimum	Maximum
Daily crash frequency	0.0266	0.1908	0.0000	10.0000
Daily average speed gap (measured as the difference between the speed limit and daily average traffic speed, in miles per hour)	59.3222	5.2399	30.7746	65.0000
Daily traffic volume (in 1000 vehicles per day)	15.4120	6.7411	0.7200	57.9568
Roadway segment length (in miles)	1.0725	0.7123	0.3680	3.6840
Inside shoulder width indicator (one if inside shoulder width is larger than five feet, zero otherwise)	0.1551	0.3620	0.0000	1.0000
Long remaining service life of rut indicator (one if the value of RUTI is higher than 99, zero otherwise)	0.2043	0.4032	0.0000	1.0000
The indexed value of the international roughness index (lower values equal rougher roads)	93.9606	5.2653	80.0000	100.0000
Daily minimum visibility (in miles)	0.8243	0.3679	0.0000	1.1000
Ratio of snowing status in the day	0.0994	0.2183	0.0000	1.0000
Ratio of wet road surface in the day	0.0946	0.1916	0.0000	1.0000
Ratio of chemical wet road surface in the day	0.0589	0.1772	0.0000	1.0000
Ratio of icy warning road surface in the day	0.1055	0.2443	0.0000	1.0000

RUTI: ruti index.

**Table 2:** Random effect hurdle negative binomial model estimation results.

Variable	Estimate Coefficients	*t*-Statistic	*p* Value
**Count state as negative binomial model**			
Constant	−9.681	−20.08	<0.0001
*Roadway Characteristics*			
Segment length (in miles)	0.533	2.63	0.0099
Inside shoulder width indicator (1 if inside shoulder width is larger than 5 feet, 0 otherwise)	1.099	2.50	0.0139
Long remaining service life of rut indicator (1 if the value of RUTI is higher than 99, 0 otherwise)	1.599	3.79	0.0003
*Weather/surface Characteristics*			
Ratio of snowing status in the day	1.253	2.98	0.0037
**Zero state as logistic model**			
*Roadway Characteristics*			
Segment length (in miles)	−0.625	−4.98	<0.0001
The indexed value of the international roughness index (lower values equal rougher roads)	0.063	23.71	<0.0001
*Weather/surface Characteristics*			
Ratio of wet road surface in the day	−0.951	−4.95	<0.0001
Ratio of chemical wet road surface in the day	−0.973	−5.32	<0.0001
Ratio of icy warning road surface in the day	−0.726	−4.46	<0.0001
Daily minimum visibility	0.231	1.81	0.0731
*Traffic Characteristics*			
Daily average speed gap (measured as the difference between the speed limit and daily average traffic speed, in miles per hour)	−0.103	−12.11	<0.0001
Daily traffic volume (in 1000 vehicles per day)	−0. 030	−4.58	<0.0001
*σ_i_*	0.554	2.23	0.0281
$\psi_{i}$	0.763	8.89	<0.0001
α	662.87	912556	<0.0001
**Summary Statistics**			
Number of observations	29462		
Log-likelihood at convergence	−3144.443		
AIC	6320.9		

*σ_i_* and $\psi_{i}:$ Random effect parameters; α: additional estimable coefficient; AIC: Akaike information criterion.
